# Postoperative Radiographic Reports After Anterior Cruciate Ligament Reconstruction: Are We Assessing What Really Matters?

**DOI:** 10.3390/jcm15113992

**Published:** 2026-05-22

**Authors:** Julien Behr, Mohamad Moussa, Nicolas Lefevre, Matthieu Sanchez, Alexandre Thomazi, Alexandre Hardy, Thibaut Noailles

**Affiliations:** 1 Department of Orthopaedic Surgery, CHU de Nantes, 1 Place Alexandre Ricordeau, 44000 Nantes, France; 2 Department of Orthopaedic Surgery, CH de Selestat, 23 Avenue Pasteur Pompidou, 67210 Selestat, France; mhamadmoussa71976798@gmail.com (M.M.); alexandre.hardy@me.com (A.H.); 3 Department of Orthopaedic Surgery, Clinique du Sport, 36 Boulevard Saint Marcel, 75005 Paris, France; docteurlefevre@gmail.com; 4 Department of Orthopaedic Surgery, Polyclinique de Bordeaux Nord, 15 rue Claude-Boucher, 33000 Bordeaux, France; docteurmsanchez@gmail.com (M.S.); a.thomazi@irsbc-kine.com (A.T.); noaillesthibaut@yahoo.fr (T.N.)

**Keywords:** anterior cruciate ligament, ACL reconstruction, radiology report, postoperative imaging, bone tunnel positioning, musculoskeletal radiology

## Abstract

**Background/Objectives:** Anterior cruciate ligament reconstruction, often combined with anterolateral ligament reconstruction, requires accurate anatomical positioning of bone tunnels, which is a key determinant of surgical success. In current clinical practice, postoperative radiographs are routinely performed for both medico-legal and clinical purposes. The objective of this study is to analyze the content of early postoperative radiology reports following ACL ± ALL reconstruction and assess their clinical relevance regarding tunnel positioning, as well as the terminology used to describe surgical findings. **Methods:** A retrospective bicentric descriptive study was conducted, including 100 consecutive postoperative radiographic reports performed within two weeks after ACL ± ALL reconstruction. The primary outcome was the mention of bone tunnel positioning. Secondary outcomes included the reporting of joint effusion, surgical complications, fixation devices, and terminology used. All radiographs were independently reviewed by two experienced orthopedic surgeons. **Results:** Among the 100 reports analyzed, interpretations were performed by 60 different radiologists. None of the reports included an assessment of bone tunnel positioning based on validated anatomical criteria. Joint effusion was reported in 83% of cases, without specification of its physiological nature. In 22% of reports, bone tunnels were not mentioned. Terms such as “sequelae” or “stigmata” of ligament reconstruction were used in 50% of reports. No complications or surgical errors were identified upon independent radiographic review. **Conclusions:** Early postoperative radiology reports after ACL reconstruction appear standardized but often lack clinically relevant contextualization regarding tunnel-related technical aspects. Rather than advocating replacement of surgeon-led image review, our findings support clearer postoperative terminology and better contextualized reporting, including explicit acknowledgment when detailed tunnel assessment is not feasible on routine radiographs.

## 1. Introduction

Anterior cruciate ligament (ACL) reconstruction, whether performed in isolation or combined with anterolateral ligament (ALL) reconstruction, is one of the most commonly performed procedures in orthopedic sports medicine [1]. Its primary objectives are to restore anteroposterior and rotational knee stability, facilitate return to sports [2], and prevent secondary intra-articular damage such as meniscal or cartilage lesions [3,4]. Surgical techniques have evolved considerably over the past decades, with increasing emphasis on anatomical reconstruction and individualized approaches.

Among the technical factors influencing surgical outcomes, the accurate positioning of femoral and tibial bone tunnels has been consistently identified as a key determinant of graft function and long-term success [5,6,7]. In particular, femoral tunnel malposition is recognized as the leading technical cause of ACL reconstruction failure [8,9], potentially resulting in persistent instability, graft overload, and early degenerative changes. As a result, postoperative evaluation of tunnel positioning has become a critical component of quality control in ACL surgery.

In current clinical practice, postoperative radiographs are routinely prescribed following ACL reconstruction, both for medico-legal documentation and for clinical assessment. These imaging studies are expected to confirm appropriate tunnel placement, verify the positioning of fixation devices, and detect early complications. However, despite their widespread use, there is no universally accepted standard for acquisition protocols or reporting practices in this specific context.

Although advanced imaging modalities such as computed tomography or magnetic resonance imaging may provide a more detailed postoperative assessment in selected situations, plain radiographs remain the first-line examination in routine practice because of their availability, low cost, and ability to document tunnel trajectories, fixation devices, and major complications. They are therefore expected to provide a pragmatic assessment of technical adequacy in the immediate postoperative period. In addition, early radiographs often represent the only imaging examination systematically available for all patients after surgery, making their interpretation particularly important from both a clinical and quality-control perspective.

Previous studies have highlighted discrepancies between the expectations of referring clinicians and the actual content of radiology reports, with clinicians generally favoring concise, clinically oriented, and actionable interpretations, whereas radiology reports often remain more descriptive and less focused on procedure-specific objectives [10,11,12,13]. In addition, increasing patient access to radiology reports has raised concerns regarding readability, clarity, and the use of terminology that may be misunderstood or unnecessarily alarming outside the appropriate clinical context [13,14,15,16]. Structured reporting has been proposed as one possible way to improve the consistency, clarity, and clinical usefulness of radiological communication [17].

These issues may be particularly relevant in the postoperative assessment of ACL reconstruction, with or without ALL reconstruction, because postoperative radiographs are not performed solely to identify rare complications, but also to verify whether key technical objectives—especially appropriate tunnel positioning—have been achieved. In this setting, a report limited to generic postoperative findings may fail to address the elements that are most relevant to the surgeon and may contribute little to postoperative quality assessment.

In this context, the issue is not whether radiology reports should replace expert postoperative surgical assessment, but whether they provide clinically meaningful and appropriately contextualized information in routine care.

Despite the recognized importance of postoperative imaging after ACL reconstruction, the actual content, focus, and clinical relevance of routine early postoperative radiology reports remain insufficiently documented. To our knowledge, few studies have specifically evaluated whether such reports address key technical parameters such as bone tunnel positioning or whether they mainly emphasize non-specific expected postoperative findings.

Therefore, the aim of this study was to evaluate the content of early postoperative radiology reports after ACL reconstruction with or without ALL reconstruction. The primary objective was to assess whether bone tunnel positioning was reported using validated anatomical criteria. Secondary objectives included analysis of the reported findings, the terminology used, and their potential clinical relevance in routine postoperative care.

## 2. Materials and Methods

### 2.1. Study Design and Setting

This retrospective bicentric descriptive study was conducted between November 2025 and January 2026 in two specialized orthopedic centers in France: Clinique du Sport, Paris, and Polyclinique Bordeaux Nord Aquitaine, Bordeaux. The study was designed to reflect routine postoperative practice and to evaluate the real-world content of radiology reports on ACL reconstruction with or without ALL reconstruction.

In both centers, postoperative care and radiographic prescription were part of routine clinical practice. Although the overall surgical philosophy was similar, the study was intentionally conducted under real-world conditions, and some radiographs were performed outside the surgical centers, reflecting usual practice.

In accordance with French regulations governing retrospective research based on anonymized pre-existing clinical data, this study did not require individual institutional review board approval. Both participating institutions had declared compliance with the CNIL reference methodology MR-004 for research not involving direct patient intervention and based on the secondary use of health data. The study was conducted in accordance with the Declaration of Helsinki and applicable national data protection regulations.

All patients were informed that their anonymized clinical data could be used for research purposes and did not object to participation.

### 2.2. Patient Selection

Consecutive patients were considered eligible if postoperative knee radiographs and their corresponding radiology reports were available within 2 weeks after primary anterior cruciate ligament (ACL) reconstruction with or without anterolateral ligament (ALL) reconstruction. Consecutive inclusion was used to reduce selection bias and to reflect routine postoperative practice.

Inclusion criteria were as follows:

- primary ACL reconstruction with or without ALL reconstruction;
- hamstring tendon autograft reconstruction using the institutional standard surgical approach;
- postoperative radiographs performed within 2 weeks after surgery;
- availability of a written radiology report for analysis.

Exclusion criteria were as follows:

- revision ACL reconstruction;
- multi-ligament knee reconstruction;
- intraoperative cartilage damage greater than grade 2 according to the International Cartilage Repair Society (ICRS) classification;
- empty, missing, or non-interpretable radiology reports.

Radiology reports and corresponding radiographs were collected during routine postoperative follow-up by an independent observer who was not involved in the surgical procedures.

### 2.3. Surgical Technique

All procedures were performed using a standardized anatomical technique [18]. ACL reconstruction was carried out arthroscopically using a hamstring tendon autograft. Femoral tunnel drilling was performed using an anatomical approach, and tibial tunnel positioning aimed to reproduce native ACL insertion sites. When indicated, ALL reconstruction was performed using a gracilis tendon graft.

Tibial fixation was achieved using a bioabsorbable interference screw (FastThread^®^, Arthrex, Naples, FL, USA), while femoral fixation relied on a radiopaque cortical suspensory device (TightRope^®^, Arthrex, Naples, FL, USA). This standardized approach ensured homogeneity of surgical procedures across the study population.

### 2.4. Radiographic Acquisition

In both participating centers, early postoperative knee radiographs were routinely prescribed by the operating surgeon as part of standard follow-up after ACL reconstruction with or without ALL reconstruction. In both institutions, the prescription requested weight-bearing anteroposterior and strict lateral knee views. The purpose of this examination was to document the postoperative appearance, assess bone tunnel trajectory and fixation devices, and identify any early obvious complication.

Although the radiographic prescription was similar in both centers, image acquisition was not fully standardized at the technical level. Radiographs could be performed either within the surgical centers or in external radiology facilities, depending on local organization and patient pathway. As a result, some variability in acquisition conditions was expected, particularly regarding strict lateral positioning, image quality, and adherence to the requested views.

This pragmatic approach was intentionally retained because the aim of the study was to evaluate radiology reporting as encountered in routine clinical practice rather than under controlled imaging conditions. Therefore, the study reflects real-world heterogeneity in postoperative imaging pathways across the two participating centers and affiliated external imaging providers.

**Figure 1:**
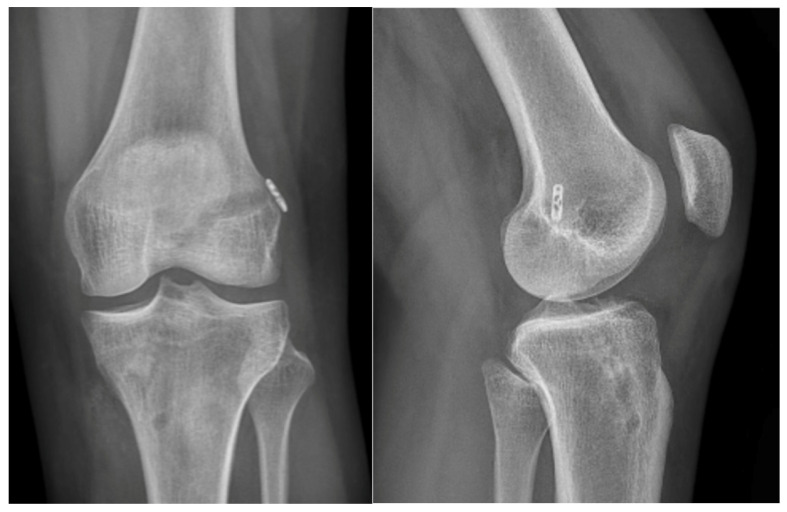
Standard postoperative radiographs after ACL + ALL reconstruction. (**Left**) Weight-bearing anteroposterior view. (**Right**) Strict lateral view.

An example of standard postoperative radiographs after ACL and ALL reconstruction, including weight-bearing anteroposterior and strict lateral views, is presented in Figure 1.

### 2.5. Radiographic and Report Analysis

All radiographs were independently reviewed by two experienced orthopedic surgeons specialized in knee surgery, each with substantial expertise in ACL reconstruction. Both reviewers were blinded to the content of the radiology reports.

The radiographic evaluation focused on bone tunnel positioning using validated anatomical criteria.

All radiographs were independently reviewed by two experienced orthopedic surgeons specialized in knee surgery, both with substantial expertise in ACL reconstruction. Both reviewers were blinded to the content of the original radiology reports during the image review.

Radiographic evaluation focused on bone tunnel positioning using validated anatomical criteria, particularly those described by Aglietti et al. [19], and derived from conventional postoperative ACL radiographic assessment, including the projected relationship between the femoral tunnel and the Blumensaat line on the lateral view, the sagittal depth of the femoral tunnel, the apparent position of the tibial tunnel, the orientation of the tunnels, the visibility and positioning of fixation devices, and the presence of any obvious postoperative complication.

In a second step, the written radiology reports were independently analyzed by the same two reviewers according to predefined criteria. The following items were specifically recorded: explicit mention of bone tunnels, explicit assessment of tunnel positioning, description of fixation devices, mention of joint effusion, characterization of effusion as physiological or pathological, mention of complications, and use of potentially misleading postoperative terminology such as “stigmata” or “sequelae.”

Inter-observer agreement for the main categorical variables was assessed using Cohen’s kappa coefficient. Disagreements between the two reviewers were recorded and subsequently resolved through joint review and consensus discussion. The final dataset used for analysis corresponded to the post-consensus assessment.

### 2.6. Outcome Measures

The primary outcome was the presence of an explicit assessment of bone tunnel positioning in the radiology report based on validated anatomical criteria.

Secondary outcomes included the mention of bone tunnels, the description of fixation devices, the reporting of joint effusion, its characterization as physiological or pathological, the identification of postoperative complications, and the terminology used to describe postoperative findings.

Potentially misleading terminology was defined as any term suggesting persistent pathological changes in a normal postoperative context, such as “stigmata” or “sequelae,” when used without appropriate temporal contextualization.

The selection of these outcomes was based on their anticipated clinical relevance in the early postoperative setting. Bone tunnel positioning was considered the primary outcome because it directly reflects surgical execution and has been repeatedly identified as a determinant of graft function and failure risk [5,6,7,8,9]. The reporting of fixation devices and complications was also considered clinically meaningful, as these elements may influence immediate postoperative surveillance. By contrast, isolated mention of effusion or unspecific postoperative changes was analyzed in order to determine whether reports tended to emphasize expected findings over surgically actionable information.

### 2.7. Statistical Analysis

A descriptive statistical analysis was performed. Categorical variables were reported as absolute values and percentages. Continuous variables were expressed as mean ± standard deviation when appropriate.

Inter-observer agreement for the main categorical variables assessed independently by the two reviewers was evaluated using Cohen’s kappa coefficient.

Given the descriptive objective of the study, no formal sample size calculation was performed and no inferential comparative statistical analysis was planned. The sample of 100 reports was considered sufficient to provide an overview of routine radiology reporting practices in this clinical setting.

All data were anonymized prior to analysis and processed using Microsoft Excel^®^ (Microsoft, Redmond, WA, USA).

This manuscript was prepared in accordance with the STROBE statement for observational studies (Supplementary Materials Table S1).

## 3. Results

### 3.1. Patient Characteristics

A total of 100 postoperative radiology reports were analyzed, corresponding to examinations interpreted by 60 different radiologists, reflecting a high variability in reporting practices.

**Figure 2:**
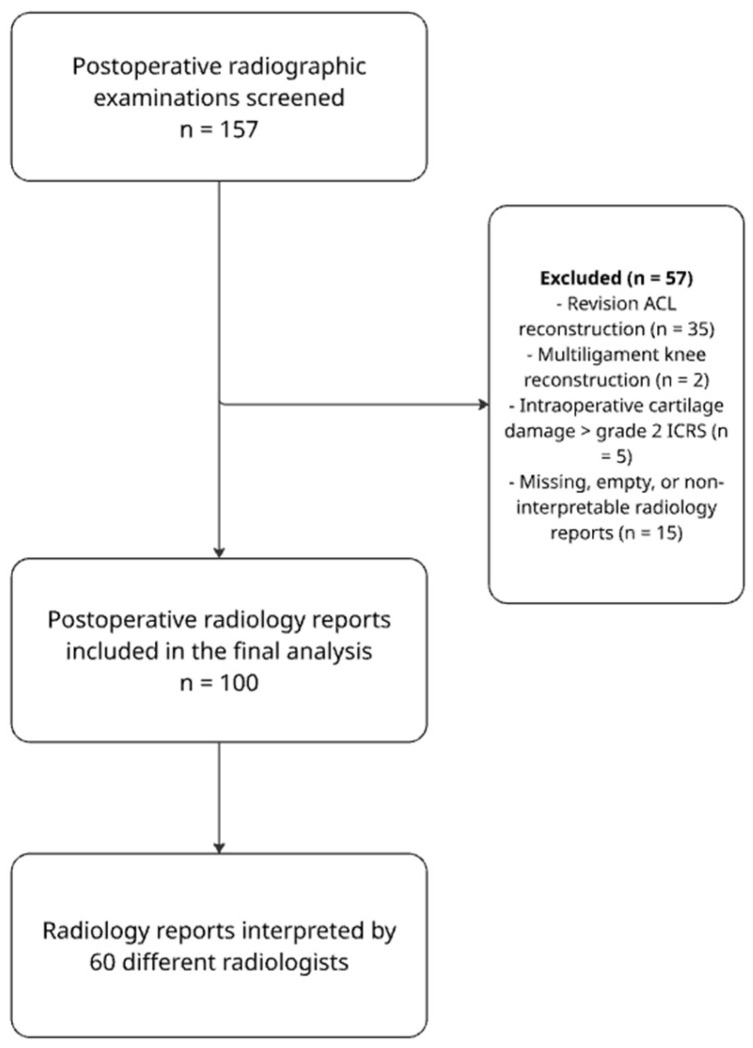
Flow diagram of study selection.

A total of 157 postoperative radiographic examinations were screened for eligibility during the study period. Among them, 57 were excluded for the following reasons: 35 flr revision ACL reconstruction, 2 for multiligament knee reconstruction, 5 for intraoperative cartilage damage greater than grade 2 according to the International Cartilage Repair Society classification, and 15 for missing, empty, or non-interpretable radiology reports (Figure 2).

After application of the eligibility criteria, 100 postoperative radiology reports were included in the final analysis. These examinations were interpreted by 60 different radiologists, reflecting substantial variability in routine reporting practice.

The demographic and surgical characteristics of the included patients are summarized in Table 1.

### 3.2. Inter-Observer Agreement

Inter-observer agreement between the two independent reviewers was substantial to almost perfect across the main categorical variables. Cohen’s kappa values were 0.98 for explicit mention of bone tunnels, 0.97 for description of fixation devices, 0.98 for mention of joint effusion, 0.89 for the use of potentially misleading terminology, and 1.00 for reported complications. Most disagreements concerned fixation device description and the interpretation of ambiguous wording related to postoperative changes. All disagreements were resolved through joint review and consensus discussion before final analysis.

### 3.3. Primary Outcome

None of the 100 radiology reports included an assessment of bone tunnel positioning based on validated anatomical criteria (0%). Independent postoperative radiographic review by the two orthopedic surgeons confirmed that no obvious tunnel malposition was present in the study cohort.

In addition, bone tunnels were not mentioned at all in 22% of reports, despite being a central technical component of the surgical procedure.

### 3.4. Secondary Outcomes

Joint effusion was reported in 83% of cases. However, none of the reports specified whether the effusion was physiological or pathological. Independent reviews indicated that the observed effusions were consistent with expected early postoperative findings and had no apparent pathological significance at this stage.

Fixation devices were described in only 9% of reports, and only 4% specifically identified the presence of a radiopaque cortical suspensory device. No abnormalities related to fixation devices were identified.

Marked variability in terminology was observed, with more than 30 different expressions used to describe postoperative findings. The terms “stigmata” or “sequelae” were used in 50% of reports, whereas other expressions such as “bone remodeling” or “postoperative changes” were used in 13% of cases.

No complications or surgical errors were reported in any of the radiology reports. This was consistent with the independent radiographic analysis, which did not identify any pathological postoperative findings.

The radiology report findings are summarized in Table 2.

## 4. Discussion

This study highlights a significant discrepancy between the expected clinical value of early postoperative radiographs after ACL reconstruction and the actual content of radiology reports. In a cohort of 100 reports, none included an assessment of bone tunnel positioning based on validated anatomical criteria [19], despite this parameter being widely recognized as a key determinant of surgical success [5,6,7].

### 4.1. Clinical Relevance of Tunnel Positioning

Accurate positioning of femoral and tibial tunnels is one of the most critical technical factors influencing outcomes after ACL reconstruction [5,6,7]. Numerous studies have demonstrated that non-anatomical tunnel placement, particularly on the femoral side, is the leading cause of graft failure and suboptimal functional outcomes [8,9]. Malpositioned tunnels may result in persistent instability, abnormal graft tension, and early degenerative changes.

In this context, postoperative imaging is expected to provide objective feedback on surgical accuracy. However, our findings suggest that this fundamental aspect is systematically overlooked in routine radiology reports. This omission raises concerns about the actual utility of these examinations in postoperative care and quality control of surgical practice.

### 4.2. Discrepancy Between Radiological Reporting and Clinical Expectations

The absence of clinically relevant information in radiology reports reflects a broader mismatch between radiologists and referring surgeons. Previous studies, including the COVER and ROVER surveys, have demonstrated that clinicians expect radiology reports to be concise, clinically oriented, and focused on actionable findings, whereas radiologists often favor descriptive and standardized reporting formats [10,13].

Our results are consistent with this discrepancy. The analyzed reports predominantly followed a descriptive approach, focusing on general postoperative findings rather than addressing specific surgical objectives such as tunnel positioning or fixation assessment. This gap may be explained by several factors, including the lack of standardized reporting guidelines, limited familiarity of radiologists with surgical techniques, and insufficient communication between surgeons and radiologists [11,12].

Several factors may account for this mismatch. First, radiologists interpreting routine postoperative radiographs may not have access to detailed operative information or may not be specifically trained to evaluate technical aspects of ligament reconstruction. Second, standard reporting habits often prioritize visible descriptive abnormalities over procedure-specific questions unless these are explicitly emphasized. Third, variability in acquisition quality, particularly when examinations are performed outside specialized centers, may discourage detailed anatomical interpretation. Together, these factors may lead to reports that are technically acceptable from a generic radiological standpoint, yet only partially aligned with the expectations of orthopedic surgeons.

The bicentric design of this study should also be considered when interpreting the findings. Although the overall surgical philosophy and postoperative radiographic prescription were similar in both institutions, the study intentionally reflected routine care conditions, including variability in local imaging pathways and the fact that some examinations were performed in external radiology facilities. This heterogeneity may have influenced image quality, strict lateral positioning, and ultimately the interpretability of postoperative radiographs. At the same time, it enhances the pragmatic value of the study by capturing reporting practices as they occur in real-world clinical settings rather than under idealized standardized conditions.

### 4.3. Interpretation of Postoperative Findings and Risk of Miscommunication

Another important finding of this study is the frequent reporting of joint effusion without contextual interpretation. While joint effusion was mentioned in 83% of reports, none specified its physiological nature. However, the literature shows that joint effusion in the early weeks following ACL reconstruction is common and often consistent with normal postoperative evolution [16]. Some studies have even reported the persistence of joint effusion several months after surgery [17], highlighting the temporal variability of this biological response.

In our cohort, independent radiographic review allowed all observed effusions to be interpreted as physiological at the given postoperative stage, with no clinical relevance. Therefore, the isolated mention of this finding, without appropriate contextualization, appears to have limited decision-making value.

This issue is further compounded by the frequent use of terms such as “stigmata” or “sequelae,” which were present in 50% of reports. Although technically correct, these terms may carry a negative connotation for patients. Several studies have demonstrated that the understanding of radiology reports differs significantly between radiologists, clinicians, and patients [13,15,16]. In a context where patients increasingly have direct access to imaging results, the terminology used becomes particularly important and is often underestimated by clinicians. Rosenkrantz et al. notably highlighted discrepancies in the perception of terminology used in radiology reports [16].

This aspect deserves particular attention in contemporary practice. With increasing transparency and digital access to test results, radiology reports are no longer read exclusively by physicians. Patients may consult their reports before postoperative review and interpret isolated terms without the necessary clinical explanation. In this setting, wording that is imprecise, overly technical, or unintentionally pejorative may generate concern disproportionate to the actual clinical situation. A report that mentions “sequelae” or “stigmata” after recent surgery may be interpreted by some patients as evidence of damage or failed healing, even when the radiographic appearance is entirely expected. Therefore, reporting language should not only be accurate, but also appropriately contextualized to avoid unnecessary anxiety and misunderstanding [13,14,15,16].

### 4.4. Need for Structured and Clinically Oriented Reporting

The findings of this study support the need for radiology reports that are better contextualized to the postoperative setting after ACL reconstruction [20].

In our view, the primary objective is not to require all radiologists to provide a definitive technical judgment on tunnel placement, but rather to improve the clinical relevance and clarity of postoperative reporting. Structured reporting has been shown to improve clarity, completeness, and communication between healthcare professionals, while also enhancing patient understanding [13].

In practice, a more useful report could systematically confirm the postoperative context, describe fixation devices when visible, mention expected early findings such as joint effusion with appropriate contextualization, and avoid ambiguous or potentially misleading terminology. When radiographic quality, surgical context, and reader expertise allow, an oriented comment on tunnel visibility or apparent positioning may be helpful. Conversely, when such assessment cannot be performed reliably on routine radiographs, this limitation should be stated explicitly.

Structured reporting, therefore, should not be understood as transferring surgical judgment from the orthopedic surgeon to the radiologist. Rather, it should be seen as a way to improve communication, reduce ambiguity, and distinguish between findings that are interpretable, findings that are expected, and findings that require surgeon-led postoperative image review.

Importantly, the objective of structured reporting is not to transfer postoperative surgical assessment from the orthopedic surgeon to the radiologist, nor to imply that routine radiology reports can replace expert surgeon-led image review. Rather, the goal is to improve the relevance and safety of radiological communication by ensuring that postoperative findings are accurately contextualized and that the limits of radiographic assessment are clearly acknowledged.

### 4.5. Clinical Implications

These findings have important implications for daily practice. For radiologists, they highlight the importance of adapting postoperative reports to the clinical context, avoiding non-specific or potentially misleading wording, and clearly acknowledging when technical assessment is limited on routine radiographs. For orthopedic surgeons, the study reinforces that postoperative image review remains a core component of surgical follow-up and should not rely exclusively on the wording of the radiology report. Improved communication between radiologists and surgeons may help ensure that imaging reports provide useful support without overstating the certainty or scope of radiographic interpretation. An example of a proposed structured radiology report is presented in Table 3.

These implications are likely to be relevant not only in specialized sports surgery centers, but also in more heterogeneous imaging networks where postoperative examinations are frequently interpreted outside the operating institution.

### 4.6. Future Directions

Future research should focus on evaluating the impact of structured radiology reporting on clinical decision-making, surgical outcomes, and patient experience. Prospective comparative studies between conventional and structured reports would be particularly valuable.

In addition, the development of consensus-based reporting templates, potentially supported by artificial intelligence tools, could help standardize practices and reduce variability. Educational initiatives aimed at improving interdisciplinary communication may also play a key role in optimizing postoperative imaging interpretation.

Beyond comparative studies, future work should also investigate the feasibility and acceptability of structured postoperative reporting in routine practice. In particular, it would be valuable to assess whether such templates are easily adopted by radiologists working in both specialized and non-specialized settings, and whether they can be implemented without increasing reporting time. Another important area of investigation would be the degree of agreement between radiologists and orthopedic surgeons when structured criteria are used for tunnel assessment. Such studies could help determine whether structured reporting not only improves report completeness, but also enhances reproducibility and interdisciplinary consistency. Finally, evaluating patient understanding of revised terminology and more contextualized conclusions could provide useful insight into the broader impact of reporting style on postoperative care.

### 4.7. Strengths

This study has several strengths. First, it addresses a practical and underexplored issue in postoperative musculoskeletal imaging, namely the actual clinical relevance of routine radiology reports after ACL reconstruction with or without ALL reconstruction. Second, the use of consecutive inclusion reduced selection bias and allowed the study to reflect daily clinical practice. Third, the bicentric design and the involvement of 60 different radiologists increased the ecological validity of the findings by capturing heterogeneous real-world reporting habits rather than the practices of a single specialized imaging team. Finally, the combination of written report analysis with independent expert review of the radiographs by two experienced knee surgeons strengthened the clinical interpretation of the results.

### 4.8. Limitations

This study has several limitations. First, its retrospective and descriptive design does not allow assessment of causal relationships or of the direct impact of radiology report content on clinical outcomes, postoperative decision-making, or patient experience. Second, the study was conducted in two specialized orthopedic centers, which may limit generalizability to other healthcare settings. However, because radiographs were obtained both within the participating institutions and in external radiology facilities, the study also reflects a broad range of routine imaging conditions.

Another limitation is the variability in radiographic acquisition, particularly regarding image quality and strict lateral positioning, as no fully standardized technical acquisition protocol was enforced across all imaging providers. This heterogeneity may have influenced the visibility and interpretability of some postoperative findings. In addition, the analysis focused on the written content of radiology reports and did not explore the radiologists’ reasoning, access to operative information, or familiarity with ACL and ALL reconstruction techniques.

The qualitative assessment of terminology also involves a degree of subjectivity, although predefined criteria, independent review, and formal inter-observer agreement analysis were used to improve reproducibility. Finally, the study did not evaluate how surgeons actually used the written reports in postoperative care, nor how patients interpreted the terminology used in these reports. These questions would deserve dedicated prospective investigation.

An additional limitation is that the study was conducted in the specific context of primary ACL reconstruction using a relatively homogeneous surgical philosophy, whereas real-world postoperative imaging may concern a broader spectrum of techniques, including revision procedures, alternative tunnel strategies, and different fixation constructs. This technical diversity may further limit the feasibility of standardized radiology reporting focused on tunnel assessment. Moreover, no universally accepted global standard exists for tunnel positioning assessment on routine postoperative radiographs, which should be taken into account when interpreting our proposed reporting perspective.

## 5. Conclusions

This study demonstrates that early postoperative radiology reports following ACL reconstruction, with or without ALL reconstruction, rarely address tunnel-related technical aspects and often emphasize non-specific postoperative findings without sufficient contextualization. In addition, some frequently used terms may be unclear or potentially misleading for both clinicians and patients.

Rather than suggesting that routine radiology reports should replace surgeon-led postoperative image assessment, our findings support a more cautious and clinically contextualized approach to reporting. Clearer terminology, better identification of expected postoperative findings, and explicit acknowledgment of when detailed tunnel assessment is not feasible on routine radiographs may improve communication between radiologists, surgeons, and patients.

## Figures and Tables

**Table 1:** Demographic and surgical characteristics of the included patients (n = 100).

Characteristic	Value
Age, years	25.9 ± 8.7
Male sex, n (%)	62 (62%)
Female sex, n (%)	38 (38%)
Right side, n (%)	59 (59%)
Left side, n (%)	41 (41%)
Time from surgery to radiograph, days	7 ± 3.2
ACL reconstruction alone, n (%)	18 (18%)
ACL + ALL reconstruction, n (%)	82 (82%)
Associated meniscal procedure, n (%)	24 (24%)
Center 1 (Bordeaux), n (%)	61 (61%)
Center 2 (Paris), n (%)	39 (39%)

**Table 2:** Summary of evaluated criteria in postoperative radiology reports (n = 100).

Evaluated Criterion	Number of Reports (n = 100)	Percentage (%)
Assessment of bone tunnel positioning	0	0%
No mention of bone tunnels	22	22%
Description of fixation devices	9	9%
Use of “stigmata” or “sequelae”	50	50%
Use of other terms (bone remodeling, postoperative changes.)	13	13%
Mention of joint effusion	83	83%
Specification of physiological nature of effusion	0	0%
Reported complications	0	0%

**Table 3:** Proposed structured radiology report for early postoperative radiographs after anterior cruciate ligament (ACL) reconstruction.

Section	Content
Examination	Postoperative knee radiographs (weight-bearing anteroposterior and lateral views)
Indication	Postoperative assessment following ACL ± ALL reconstruction
Bone tunnel positioning	Description of tunnel visibility and postoperative appearance; when image quality, surgical context, and reader expertise allow, a comment on apparent tunnel positioning may be provided. If precise assessment is not reliable on routine radiographs, this should be stated clearly.
Fixation devices	Description and positioning of fixation devices (e.g., interference screw, cortical suspensory device)
Joint effusion	Presence of joint effusion, with specification of its expected physiological nature in the early postoperative period
Bone and joint structures	Assessment of bone integrity and absence of abnormal findings
Complications	Detection of early complications (e.g., hardware failure, fracture, abnormal tunnel position)
Conclusion	Concise summary focusing on clinically relevant postoperative findings and clearly stating whether tunnel assessment is feasible on the available radiographs and absence/presence of complications

## Data Availability

The data presented in this study are available from the corresponding author upon reasonable request.

## Supplementary Materials

The following supporting information can be downloaded at https://www.mdpi.com/article/10.3390/jcm15113992/s1, Table S1: STROBE Checklist.
