# Supplementary material for: Postoperative Radiographic Reports After Anterior Cruciate Ligament Reconstruction: Are We Assessing What Really Matters?
Source: J Clin Med. 2026 May 22;15(11):3992. doi: 10.3390/jcm15113992 (PMC13257830; doi:10.3390/jcm15113992)
Supplement: Supplementary file 1 [file jcm-15-03992-s001.zip › jcm-4239593-supplementary.pdf]

**Table S1.** STROBE Checklist.

| Section / Topic | Item | STROBE recommendation | Location in manuscript |
|---|---|---|---|
| TITLE AND ABSTRACT | 1a | Indicate the study's design with a commonly used term in the title or the abstract | Abstract; Materials and Methods – Study design and setting |
| | 1b | Provide in the abstract an informative and balanced summary of what was done and what was found | Abstract |
| INTRODUCTION | 2 | Explain the scientific background and rationale for the investigation being reported | Introduction |
| | 3 | State specific objectives, including any prespecified hypotheses | End of Introduction |
| METHODS | 4 | Present key elements of study design early in the paper | Materials and Methods – Study design and setting |
| | 5 | Describe the setting, locations, and relevant dates, including periods of recruitment, exposure, follow-up, and data collection | Materials and Methods – Study design and setting |
| | 6 | Give the eligibility criteria, and the sources and methods of selection of participants | Materials and Methods – Patient Selection |
| | 7 | Clearly define all outcomes, exposures, predictors, potential confounders, and effect modifiers | Materials and Methods – Outcome measures |
| | 8 | For each variable of interest, give sources of data and details of methods of assessment | Materials and Methods – Radiographic and report analysis |
| | 9 | Describe any efforts to address potential sources of bias | Materials and Methods – Patient Selection; Study design and setting |
| | 10 | Explain how the study size was arrived at | Materials and Methods – Statistical analysis |
| | 11 | Explain how quantitative variables were handled in the analyses | Materials and Methods – Statistical analysis |
| | 12 | (a)Describe all statistical methods, including those used to control for confounding<br>(c)Explain how missing data were addressed | Materials and Methods – Statistical analysis<br><br>Results – Study population and selection flow |
| RESULTS | 13 | (a)Report numbers of individuals at each stage of study<br>(b)Give reasons for non-participation at each stage<br>(c)Consider use of a flow diagram | Results – Study population and selection flow<br>Figure 2 |
| | 14 | Give characteristics of study participants and information on exposures and potential confounders<br>Indicate number of participants with missing data for each variable of interest | Results – Patient characteristics; Table 1<br><br>Results – Study population and selection flow |
| | 15 | Report numbers of outcome events or summary measures | Results – Primary outcome; Secondary outcomes; Table 2 |
| | 16 | (a)Give unadjusted estimates and, if applicable, confounder-adjusted estimates and their precision<br>(b)Report category boundaries when continuous variables were categorized | Not applicable |
| | 17 | Report other analyses done, such as subgroup analyses and sensitivity analyses | Inter-observer agreement |
| DISCUSSION | 18 | Summarize key results with reference to study objectives | Discussion ; Conclusion |
| | 19 | Discuss limitations of the study, taking into account sources of potential bias or imprecision | Discussion – Limitations |
| | 20 | Give a cautious overall interpretation of results considering objectives, limitations, multiplicity of analyses, results from similar studies, and other relevant evidence | Discussion |
| | 21 | Discuss the generalizability of the study results | Discussion – bicentric design / Limitations |
| OTHER INFORMATION | 22 | Give the source of funding and the role of the funders | Funding |
